# A focus group study of therapists’ views on using a novel neuroanimation virtual reality game to deliver intensive upper-limb rehabilitation early after stroke

**DOI:** 10.1186/s40945-022-00139-0

**Published:** 2022-06-15

**Authors:** Rachel C. Stockley, Danielle L. Christian

**Affiliations:** Stroke Research Team, Faculty of Health and Wellbeing, University of Central Lancashire, PrestonPreston, PR1 2HE UK

**Keywords:** Focus groups, Rehabilitation, Stroke, Upper extremity, Video games

## Abstract

**Background:**

Intensive training can significantly reduce upper-limb impairments after stroke but delivering interventions of sufficiently high intensity is extremely difficult in routine practice. The MindPod Dolphin® system is a novel neuroanimation experience which provides motivating and intensive virtual reality based training for the upper-limb. However several studies report that health professionals have reservations about using technology in rehabilitation. Therefore, this study sought to explore the views of therapists who had used this novel neuroanimation therapy (NAT) in a clinical centre to deliver intensive for the upper-limb of people after stroke in a phase 2 trial (SMARTS2).

**Methods:**

Four therapists (three female, two physical and two occupational therapists) who delivered NAT participated in a focus group conducted by two independent researchers. The theoretical domains framework and COM-B behaviour change models informed the discussion schedule for the focus group. An inductive approach to content analysis was used. Recordings were transcribed, coded and thematically analysed. Generated key themes were cross-checked with participants.

**Results:**

Whilst therapists had some initial concerns about using NAT, these were reduced by training, reference materials and face-to-face technical support. Therapists noted several significant benefits to using NAT including multi-system involvement, carry-over to functional tasks and high levels of patient engagement.

**Conclusions:**

These findings illuminate key areas that clinicians, technology developers and researchers should consider when designing, developing and implementing NAT. Specifically, they highlight the importance of planning the implementation of rehabilitation technologies, ensuring technologies are robust and suggest a range of benefits that might be conferred to patients when using intensive NAT as part of rehabilitation for the upper-limb after stroke.

**Supplementary Information:**

The online version contains supplementary material available at 10.1186/s40945-022-00139-0.

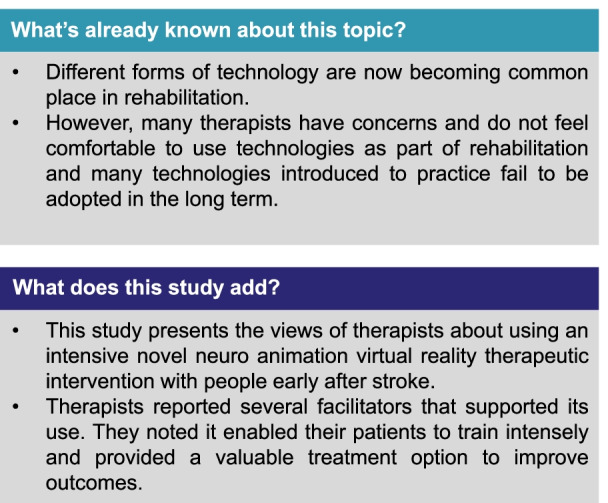



## Background

Over 15 million people have a stroke each year worldwide and stroke is the single main cause of acquired disability in high income countries [1, 2]. Difficulty using the upper-limb is the most common deficit after stroke and is reported by over 70% of stroke survivors [3, 4]. Less than 20% of people after stroke recover full upper-limb function and over half have not regained basic functions of the upper-limb after several years [3–5]. Consequently, improving the recovery of the upper-limb is a focus of both stroke rehabilitation and research [6–8].

In animal studies, the quality of movements and the intensity of training significantly influence the recovery of limb movements after stroke [9]. In humans, it is suggested that at least several hundred movement repetitions per treatment session in the first weeks after stroke is likely to optimise benefits [10–16]. This intensity (the amount of work per episode or time)[17] has been notoriously difficult to deliver within conventional therapy. The upper-limb is reported to be the focus of treatment for 29 min three times a week and an average of only 30 repetitions are delivered in typical rehabilitation sessions in reports from the UK and Australia [18, 19].

Novel neuroanimation therapy (NAT) requires patients to make a large number of high-quality movements in three dimensions using their upper limb in order to play an immersive, enjoyable bespoke video game. This represents an attractive and engaging medium by which therapists can offer motivating, high dose therapy [20]. However, implementation of technologies can be difficult. A survey found only 23% of 505 American of clinicians felt very or extremely knowledgeable about rehabilitation technologies (e.g. wearable sensors, tablets) and about half of occupational and physical therapists were not comfortable to integrate technologies into their practice [21]. Others have reported that using video games (which have several similarities to NAT) as an integral part of rehabilitation has practical barriers [22], can cause health professionals to question their role and elicit a perceived lack of control over therapy progression [23, 24]. These concerns may become amplified when using NAT to deliver particularly intensive therapy but this has not been explored as no studies have implemented NAT in this way. Therefore, the aims of this study were to: (1) describe the experiences of therapists when using NAT to deliver intensive therapy for the upper-limb for people in the early acute period after stroke and, (2) identify barriers and facilitators to its use.

## Methods

### The intervention

**Fig. 1:**
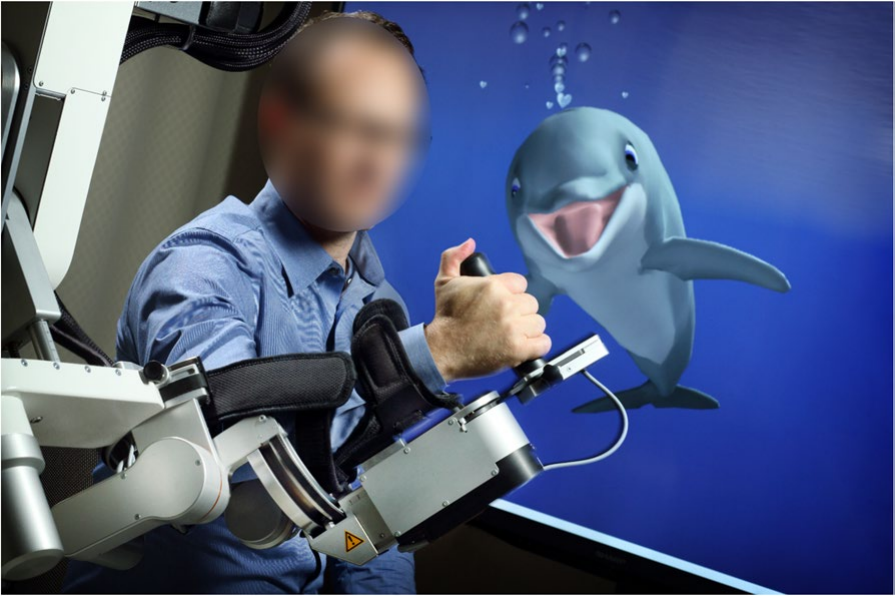
Video game training system used in the SMARTS trial comprising the MindPod Dolphin® video game with Armeo system

A recent phase II randomised, single-blinded, pilot multicentre trial, Study to enhance Motor Acute Recovery with intensive Training after Stroke, (SMARTS 2; NCT02292251) [25] utilised intensive (one hourly twice daily sessions, five days a week for three weeks) impairment-focussed, interactive NAT during the first ten weeks after stroke. A proprietary game, MindPod Dolphin® (Fig. 1) delivered NAT. The patient’s paretic arm was unweighted using the Armeo®Power (Hocoma AG, Volketswil, Switzerland), an upper limb exoskeleton device. This allowed practice of 3D arm movements despite antigravity weakness without requiring a therapist to actively lift the paretic arm. The degree of unweighting provided by the exoskeleton was adjusted for each patient to maintain shoulder flexion to 90 degrees at rest so as to provide weight-support of the paretic limb throughout its full active range in all directions. In the game, 3D movements of the paretic arm controlled the movement of a virtual dolphin (Bandit), swimming through different ocean scenes. A large screen displayed the dolphin in his environment, oceanic sounds and music were played, and the lights were dimmed for the entirety of each session. To progress the game, a patient had to control the dolphin to complete various goals including chasing and eating fish, eluding attacks, and performing jumps often within a limited time (measured by a countdown timer displayed on the screen). Progression could also be tailored by the clinician as well as an automatic incremental increase in difficulty within the game. Tasks were designed to promote movement in all planes throughout the active ranges of motion and outside of the synergistic patterns typical after stroke, thus supporting quality of movement. As the robotic exoskeleton and the video game were always used in concert, they are collectively classified as NAT for the purposes of the current study.

Four therapists administered the NAT at Johns Hopkins Hospital, Baltimore, USA in the SMARTS2 trial. They remained with each individual patient during game play, providing assistance, such as setting up the robotic arm, positioning the patient appropriately, starting the game, progressing the game when indicated and initiating rest periods where needed.

### Participants

Four therapists who constituted all the therapists who delivered NAT at one of the SMARTS2 sites were emailed and purposively recruited to explore their experiences of delivering intensive NAT with patients after the trial had finished. They had received training on using NAT including a day of face-to -face instruction, video and practical experience, ad hoc refresher training when required, and were provided with a training manual and had access to the technical developers/experts on NAT. All participants provided informed consent. Ethical approval was granted for the focus group by the University of Central Lancashire Science Technology Medicine and Health Ethics Committee (STEMH 933) and Johns Hopkins Medicine Institutional Review Board (IRB 00,203,795).

### Focus group

A focus group methodology was utilised to maximise the interaction between participants and provide insights into any shared group culture as participants already knew each other well due to the nature of their work [26]. The focus group discussion guide (Additional file 1: Appendix 1) was developed using principles of behaviour change, notably the COM-B model [27, 28], the theoretical domains framework, TDF, [29] and explored:

- Confidence, motivation and skills to use NAT (COM-B)
- Perceived and real barriers and facilitators in using NAT to deliver intensive therapy (COM-B)
- Perceptions of the effects of using NAT to deliver high intensity training for the upper-limb (TDF)
- If and how NAT could be implemented into wider practice (TDF)

The focus group was conducted via video link between the therapists based in the hospital and two experienced researchers from the UK who were not directly involved in the SMARTS2 trial (RCS and DC). No others were present during the focus group. RCS, a physiotherapist, had met the participants three times prior to the focus group and had collaborated with them on initial development of this study and is a mixed methods researcher. DC is not a healthcare professional, had not met the participants previously and is a qualitative researcher having undertaken a range of qualitative interview studies in both health and education. The interviewers shared the questioning. Specifically, RCS lead the questioning and moderated on the expectations and prior experience of using technology in rehabilitation and explored the barriers to clinical implementation after the trial. DC led the questioning and moderated on the participant’s general background and their experiences of using NAT in the trial. Two audio recorders were used to record the focus group in addition to a video recording via Skype for Business.

A semi-structured approach, initially piloted with UK research and therapist colleagues, was used to allow a more natural flow [26]. Whilst one researcher took the lead on questioning, the other observed interactions between participants and made field notes. The key points were summarised to the participants verbally at the end of the group to ensure respondent validation [30].

### Analysis

The focus group was transcribed verbatim in Microsoft Word by one researcher (DC) [31, 32]. An inductive approach to content analysis was used. Two researchers (RCS and DC) read and re-read the transcripts and independently generated and assigned codes in MS Excel [31]. Codes were compared for consistency and discrepancies were resolved by discussion and going back to the original text. Similar codes were collated to form larger themes [31]. Specific quotes from participants were collated under their respective themes. Themes and quotes were cross-checked with all participants [32].

## Results

The focus group lasted 95 min. No participant raised any issues regarding the accuracy of the themes and/or quotes during cross-checking. Participants (three females) comprised two physical (PT) and two occupational therapists (OT) who had been qualified between 4.5 to 15 years and worked in the neurosciences service for the entirety of their career. They agreed that patients tended to stay on their unit for between 4 to 7 days during which time they received rehabilitation usually on a daily basis delivered by both OT and PTs.

Codes were generated from the focus group data and three themes were identified and are summarised in Table 1 and in Additional file 2: Appendix 2.

**Table 1:** Key themes and sub-themes

Theme	Sub-themes
1. Perceived mechanism by which NAT might work	Immersion, motivation, interdependency of NAT and therapists, changing focus of treatment
2. Observed effects of intensive NAT	Carry over to better function of the upper-limb, wider physical, cognitive and visual benefits beyond the upper-limb
3. Implementation of NAT in the trial and in wider practice	Real and perceived barriers, resources and training, patient selection

### Theme 1 – Perceived mechanism by which NAT might work

Participants identified that patients found NAT immersive and motivating.

Participant (P) 1: “They kind of got lost in the game…they were much more focused on Bandit and the dolphin swimming round on the screen than they were about their arm.”

P4: “One of my favourite things was the timer and then the…motivation that it created….. to unlock the new level…”.

Participants reported that the patients’ use and experience of the game benefited from the therapists’ involvement. They highlighted that using NAT enabled patients to be challenged more effectively during therapy and commented that it allowed them to shift focus of treatment towards quality of movement rather than completion of tasks and problem solving. However, they were clear that they were using NAT as a valuable part of therapy, rather than the therapy being solely provided by the NAT.

P2: “I think I was surprised by how much the technology helped me challenge the patient more, longer, harder.”

P3: “in my mind, it doesn’t replace therapy, it just becomes therapy.”

P2: “I was surprised at how much of my skill was needed to use it. … it wasn’t just the tech that was doing the work. I felt that we were really making it what it was…”.

P2: “I think it’s such a valuable supplement and I wouldn’t leave home without it”.

### Theme 2 – Observed effects of intensive NAT

Participants discussed that NAT provided a multi-sensory challenge for the patients. They noted that although the system was impairment-focussed, there were also clear benefits to functional tasks. One participant relayed her experience of speaking to one patient’s wife who had reported a clear improvement in her husband’s condition:

P1: “…she (the patient’s wife) had come out of the bathroom and he (the patient) had already put his shoes on by himself and that was something he never was able to do at the beginning of the trial and this was mid-trial. So that was almost as reinforcing for me to be like ‘OK, we’re not just working on video gaming.’ There is a carry over. This is going other places.”

Participants also commented on the challenges and wider benefits to other systems such as vision, balance and cognition, although noted that the challenging nature of the game meant that some patients experienced more fatigue.

P1: “And improvement in other areas, such as we weren’t necessarily addressing vision or cognition but when they’re looking at a full screen …they have to visually attend to the full screen to engage in the game, we saw improvements with that, with balance, gait, there was a little bit of everything.”

P2: *“*And visually I think the game was a lot more visually stimulating than the conventional therapy so a lot of times I know that they were much more cognitively bad after the game”.

### Theme 3—Implementation of NAT in the trial and in wider practice

Participants discussed their initial confidence in using NAT and the training and resources they utilised to implement it into practice. They identified perceived barriers (concerns prior to using NAT) and real barriers experienced during implementation that could affect the usability of NAT.

P4: “I definitely always had at the back of my mind that technology could fail me and I would be stuck not being able to work the system so…[Laughs]. It did happen sometimes but we trouble-shooted and it would delay our treatment a little bit but it was OK.”

All participants noted how useful familiarisation and training was prior to using NAT with patients and identified resources including a manual and access to a technology expert that supported its ongoing successful use.

P4: “…I think we did adequate training sometimes with the whole…group and sometimes on our own together to make sure we felt comfortable.”

“…we had this really awesome book that we could refer to … for me that was very helpful.”

P2: “I think there had to be… a resource or technology expert available so there has to be somebody that knows what to do if things go wrong…”.

Participants discussed how NAT could be used in wider clinical practice and highlighted that they had significantly more time to deliver treatment for the upper-limb that they would usually, having as long as required to deliver 60 min of actual game play, because of their involvement in the trial.

P2: “So it didn’t matter how long that was actually going to take. We were gonna be in there as long as it took to get 60 min which was sort of liberating.”

They did identify several barriers related to clinical settings, but simultaneously expressed a desire to use NAT more widely.

P1: “Using it would definitely accelerate remediation I feel like. I feel like you would obviously get more repetitions, better quality…”.

Barriers to implementation in other clinical settings included space for equipment, infection control and patient awareness.

P3: “there’s patient issues, so there’s infection risk, there’s incontinence issues, there’s arousal issues for patients who’ve had more severe stroke and their ability to interact with their environment at all versus interact with a video game”.

They also noted the need to have flexible methods of delivery for different patients.

P3: “… I think that needs to be tailored to each patients’ needs. The more severely paretic or severely cognitively impaired patients probably needs to be one-on-one but some of your higher functioning patients would probably benefit from a group setting”.

## Discussion

Current guidelines encourage intensive practice of functional, task-orientated repetitive training to significantly improve the performance of everyday activities with the upper-limb [14], but recent studies suggest that intensive impairment-focussed training, specifically targeting the quality of movement, as opposed to functional task completion, may prove more beneficial [33]. The SMARTS2 trial is the first to deliver true intensive impairment-focussed NAT, finding that it was equivalent to time matched additional therapy, but significantly more beneficial than usual care [25]. The results of this qualitative study highlight some of the key perceptions, barriers and facilitators around using this kind of NAT in clinical practice and so are both significant and meaningful.

### Capability

Participants noted that familiarisation training, physical resources (a training manual) and skilled face-to-face assistance were important sources of support prior to and during the trial increasing their capability to use the technology [28]. As the self-efficacy of therapists is an important predictor of clinical uptake, the findings of this study underline the need to support considering capability in the clinical implementation of innovations to ensure that users are confident to use them [34]. Whilst the NAT system in this study encountered very few technical problems others have reported therapists’ frustrations with repeated technical problems in similar systems, resulting in a rapid decline in their perceived trustworthiness, value and ultimately precipitating their non-adoption or abandonment [34–36]. The differences between the current study and these reports highlight the need for the involvement of users in the design of rehabilitation technologies and rigorous testing prior to clinical use to ensure optimal acceptability and usability [24, 37].

### Motivation

One of the unique elements of the SMARTS2 trial was that therapists spent significantly longer focussing upon treatments for the upper-limb than during standard rehabilitation practice which may account for some of their motivation to use it [15, 19]. The participants reported that their patients could complete the intensive training provided by NAT and became quickly immersed in the game, supporting many of the well-reported reasons for using video gaming media [20, 38]. The immersion to which the therapists referred appeared to represent a sense of presence in a virtual environment, or place illusion [39]. Participants also noted the NAT using the MindPod Dolphin® provided a graded, intensive, multi-system challenge that conferred significant benefits both for the upper-limb and other systems which could not be easily replicated in conventional therapy. These benefits appeared to further motivate the therapists to continue using NAT and, according to theories of behave change, are likely to influence their willingness to use it beyond the study [28]. They valued the additional time for therapy afforded by the trial and did not express significant concerns about the intensity of NAT being harmful for their patients. Indeed, the presence of physical and cognitive fatigue, a potentially deleterious side effect of undertaking high intensity NAT, was largely perceived as a positive effect of the intervention which indicated that patients were being significantly challenged.

Participants repeatedly commented that they recognised that NAT was a useful and novel addition to, rather than a replacement for, their therapeutic skills and that it enabled a greater focus on quality of movement than would be typically possible in conventional therapy. This observation aligns with the perceived generalised usefulness of video game based interventions reported by therapists [23]. Importantly, the inherent focus on movement quality in NAT allays concerns about promotion of unwanted movements which have been reported in studies of other forms of video game training.[23].

A significant finding of this study is that participants reported that they were not passive when patients were undertaking NAT and felt that working in partnership with the technology was vital for an optimal outcome. It is possible that the perceived need for therapist involvement during NAT may be attributable to the inpatient setting and relative recency of stroke in the patients in the SMARTS2 trial. However, the findings suggest that NAT was perceived to provide novel, additional and uniquely challenging therapy, which could not be replicated in conventional therapy and so provided an innovative and valuable treatment for people after stroke.

### Opportunity

Whilst therapists only used NAT as part of the SMARTS2 trial, they did discuss how it could be used in routine practice although recognised that there was currently no specific opportunity for this to continue. They identified the additional support needed by some patients and barriers in clinical settings due to the space the system required, a commonly reported limitation to NAT use [24, 40]. Crucially, they noted that the time provided to deliver therapy solely for the upper-limb in the SMARTS2 trial far surpasses that offered in usual care which would provide a significant challenge to replicate in clinical practice [15, 19].

### Limitations

There are several limitations of this study. The sample was small (four therapists, who delivered all NAT) and based at one site of this international multi centred trial. Only one site was selected for this focus group to avoid comingling from different rehabilitation settings and healthcare systems but this does limit the generalisability of findings. The group dynamics inherent to focus groups can also result in an artificial convergence of views [31]. However, this did not seem apparent in this study as all participants contributed equally to discussions and where disagreements occurred, they were resolved without any intervention from the researchers, suggesting that participants felt comfortable to express and challenge divergent views. Whilst RCS is a physiotherapist with an interest in NAT and her views may have biased the results, DC had no experience of clinical rehabilitation or NAT, reducing the impact of any bias.

Finally, as NAT was delivered as part of a clinical research trial, some barriers and facilitators inherent to routine clinical practice did not feature in respondents’ discussions. These include the costs of equipment and the substantial time necessary to deliver intensive NAT [24] which require further elucidation to ensure that this therapy is economically plausible.

## Conclusions

This work provides detailed insights into therapists’ views on using a unique, impairment-focussed NAT system to deliver intensive therapy for the upper-limb in the early period after stroke. They reported that NAT facilitated intensive therapy and provided holistic, engaging and challenging game play which motivated patients. They expressed few concerns about the intensity of NAT and valued the increased time for treatment. Written materials, training and face-to face support to underpin the implementation of NAT were considered important as well as the reliability of the NAT system. A novel finding of this study is that therapists felt that their presence was needed in order to make the NAT optimally beneficial. Further work should now be undertaken to implement and subsequently evaluate therapists’ perceptions of using NAT to deliver intensive therapy in routine practice, to identify whether these study’s findings are replicated in a real-world setting.

## Supplementary Information

**Additional file 1. **Focus Group Schedule.

**Additional file 2. **Map of codes and themes.

## Data Availability

The full datasets generated and/or analysed during the current study are not publicly available due to reasons of confidentiality but anonymised data are available from the corresponding author on reasonable request.

## Abbreviation

NAT: Novel Neuro animation Therapy
