# Supplementary material for: A focus group study of therapists’ views on using a novel neuroanimation virtual reality game to deliver intensive upper-limb rehabilitation early after stroke
Source: Arch Physiother. 2022 Jun 15;12:15. doi: 10.1186/s40945-022-00139-0 (PMC9199178; doi:10.1186/s40945-022-00139-0)
Supplement: Supplementary file 1 — Additional file 1. Focus Group Schedule. [file 40945_2022_139_MOESM1_ESM.docx]

**FOCUS GROUP SCHEDULE**

| **AREA** | **QUESTION** | **NOTES** |
| --- | --- | --- |
| **(-)** | **Introductions and importance of speaking clearly**  **Setting of ground rules**  **Re-capping of aims**  **Definition of Video Gaming and control treatments**  **Establishing who provided control treatment and who provided VG** |  |
|  | **INTRODUCTORY QUESTIONS** |  |
| **(-)** | - Tell us about your role in stroke rehabilitation at JHU   - What is your current work setting?   - How long do you typically have to work with patients before they are discharged or moved to another service? - How long have you been working specifically in stroke rehabilitation? - Tell us about your usual treatments for the upper limb of people who have had a stroke. |  |
|  | **PRE-STUDY** |  |
| **(-)** | - How did you become involved with the SMARTS trial? Did you want to be involved in the study and use VG? Why? - Was this your first time being involved in research? |  |
| **(1)**  **(7)**  **(5)** | - Tell us about what you knew about video gaming (VG) in rehabilitation before taking part in the trial   - Had you used it before? How and when?   - Did you know how to use it?   - Were you confident in using it?   - Was VG something that was usually undertaken on your unit? - Did you think that the VG interventions would work in your clinical setting? |  |
| **(1,2)**  **(5)**  **(7)**  **(5)** | - Tell us about your original thoughts on the control treatments and how these were to be delivered before taking part in the trial   - How were these treatments chosen?   - Were they the same for everyone?   - What did you think about the effectiveness of your treatments before the study?      - - Was the control intervention something you usually did in your unit?   - Tell us about how and when you used the control treatments before the trial - Did you think that the control interventions would work in your clinical setting? |  |
|  | **DURING THE STUDY** |  |
| **(2)**  **(4)**  **(2,8)**  **(10)**  **(10)** | - Tell us how you used the VG training system in practice.   - How easy was it to use the VG gaming training system in practice?   - Did you feel capable to deliver the VG the treatment in the trial?   Why?   - Tell us about the physical resources needed to provide the VG treatment   - Were there any difficulties in using it the way it was supposed to be used?   - Were there any occasions when the VG could not be completed/there were any problems? If so, what did you do? - How did you personally feel about using VG?   - Do you think it is useful? Why? Why not?   - Do you think it can increase the dose of therapy? How? Why? Why not? - Did using VG provoke an emotional response?   - From who? What was it?   - How did emotion affect the use of VG? |  |
| **(2, 8)**  **(4)**  **(2, 8)**  **(10)**  **(10)** | - Tell us about what the control treatment comprised   - How difficult was it to deliver the control intervention in practice?   - Could you apply the treatment the way it was supposed to be used?   - Did you feel capable to deliver the control treatments in the trial?   Why?   - Tell us about the physical resources needed to provide the control treatment   - Were there any things you could not do due to time or the tasks?   - How did the time allowed for the control treatment affect its content?   - Were there any occasions when the control treatment could not be completed/there were any problems? If so, what did you do? - How did you personally feel about providing the control treatment to your patients?   - Do you think it is useful? Why? Why not?   - Do you think it can increase the dose of therapy? - Did using the control treatment provoke an emotional response?   - From who? What was it?   - How did emotion affect the use of the control intervention in rehabilitation? |  |
| **(9)** | - Tell us about who and how others influenced your use of VG (could include patient, manager, professional groups, evidence base, carers) - Tell us about who and how others influenced your use of the control treatments (could include patient, manager, professional groups, evidence base, carers) |  |
| **(3)** | - Tell us how you felt being part of the trial   - Did you have any concerns or misgivings about what you were being asked to do?   - Were the treatments you were given similar to those you would routinely provide? - Do you think what you did is different to the therapy a stroke survivor might receive if they were **not** in the trial? How? Why? |  |
| **(6)**  **(6)**  **(6)** | - Did you feel using VG conflicted with any other guidance/guidelines/practice?   - If so how did you handle that?   - In the case of conflict, what was your priority? - Did you feel using the control treatments conflicted with any other guidance/guidelines/practice?   - If so how did you handle that?   - In the case of conflict, what was your priority? - Did you have any concerns about quality of movement when using either treatment? |  |
|  | **POST-STUDY** |  |
| **(5)**  **(6)** | - Tell us about how your thoughts about using VG have changed now following the study. Recap briefly some of the thoughts from earlier.   - - What did you think about the usefulness of VG after the study?     - Did you feel the VG treatments benefitted your patient? How? Why?     - How did the patients respond to VG? |  |
| **(5)**  **(6)** | - Tell us about how your thoughts about your control treatments for the upper limb have changed now following the study.   - - What do you think about the usefulness of the control intervention now?     - Did you feel the control treatments benefitted your patient? How? Why?     - How did the patients respond to VG? |  |
| **(5)**  **(6)**  **(12)**  **(5)** | - What did/do you expect the results of the study to show? - What were the key differences between the control treatments/VG to your usual practice? - What difference and similarities do you think there were between the VG and control interventions?   - - Do you have a view of which was better? Why? Why not? - Has being the trial changed your practice in any way? If so, how? |  |
|  | **GOING FOrwARD** |  |
| **(12)**  **(11)**  **(5)**  **(12)** | - Did you want to continue to use VG system after the trial?   - If so, why? - How do you think VG could be used in your setting?   - What steps would be needed if VG was to be used more widely within your organisation?   - How could that happen practically?   - Could VG be used in a group setting? Why do think it could/couldn’t?   - Have you given much thought to the costs of using VG? - Do you think VG should be more widely available for use in rehabilitation?   - From your experiences, what needs to be done to make this happen?   - Specifically – do you think you can incorporate VG in the current time you have for therapy? |  |
| **(12)**  **(12)**  **(11)**  **(5)** | - Did you want to continue to use the control interventions after the trial?   - If so, why? - Do you think the control intervention should be more widely available for use in rehabilitation?   - From your experiences, what steps would be needed if the control intervention was to be used more widely within your organisation?   - How could that happen practically?   - Have you given much thought to the costs of using treating patients as you did in the control group? |  |
|  | **CLOSING QUESTIONS AND RECAP** |  |
|  | - Is there anything else you wish to add to the discussions? - Briefly recap the main discussion points |  |
|  | **Thank you for taking part.**  **Our next steps are to transcribe and draw out themes. These will be sent to you all for checking. If something doesn’t seem reflective of what was said or you feel we have missed something, you will be able to tell us.** |  |

NB: Main headings (indicated by a ‘-‘ bullet) are the key questions that were asked, bullets beneath (o) indicate potential prompts/specifics to ask if required.

Area key from COM B and TDF

1. Knowledge
2. Skills
3. Self-standards
4. Beliefs about capabilities
5. Beliefs about consequences
6. Motivations and goals
7. Memory, attention and decision processes
8. Environmental context and resources
9. Social influences
10. Emotion
11. Behavioural Regulation
12. Nature of the behaviours
