# Supplementary figures and images for: A focus group study of therapists’ views on using a novel neuroanimation virtual reality game to deliver intensive upper-limb rehabilitation early after stroke
Source: Arch Physiother. 2022 Jun 15;12:15. doi: 10.1186/s40945-022-00139-0 (PMC9199178; doi:10.1186/s40945-022-00139-0)

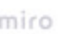

Supplement: Supplementary file 2 — Additional file 2. Map of codes and themes. [file 40945_2022_139_MOESM2_ESM.pdf]
